# Primary Rifampicin Mono-Resistant Tuberculosis of the Cervical, Mediastinal, and Axillary Lymphadenopathy in an Indian Male: A First-of-Its-Type Case

**DOI:** 10.7759/cureus.58565

**Published:** 2024-04-18

**Authors:** Sankalp Yadav

**Affiliations:** 1 Medicine, Shri Madan Lal Khurana Chest Clinic, New Delhi, IND

**Keywords:** mediastinal lymphadenopathy, axillary lymphadenopathy, cervical lymphadenopathy, cbnaat/ xpert/ rif assay, mycobacterium tuberculosis (mtb), primary rifampicin mono-resistant tuberculosis

## Abstract

Tuberculosis is common in endemic countries. However, extrapulmonary tuberculosis is relatively rare, and primary extrapulmonary rifampicin mono-resistant tuberculosis of the cervical, mediastinal, and axillary lymph nodes simultaneously without pulmonary focus in an immunocompetent male has never been reported. Herein is a case of a 27-year-old Indian male with no previous history of tuberculosis who was diagnosed after an extensive clinical assessment with a radiometric and cartridge-based nucleic acid amplification assay of the swollen lymph nodes. He was put on antituberculous treatment per the all-oral longer regimen of the national program.

## Introduction

*Mycobacterium tuberculosis* is one of the leading causes of morbidity and mortality in high-burden countries [1]. This infection is spread mainly by infected aerosols originating from a diseased person [2]. Often manifesting as pulmonary tuberculosis, extrapulmonary sites are also affected [3].

Drug-resistant tuberculosis is a constant challenge for the healthcare system [4]. Even in endemic countries, timely diagnosis and treatment initiation in these cases is an arduous task. The situation becomes even more challenging when the patient presents with no pulmonary involvement. Moreover, a delay in management could have fatal outcomes. Drug-resistant tuberculosis can be of various types, and rifampicin mono-resistant tuberculosis is a form where there is resistance to rifampicin without resistance to any other first-line antituberculous drugs [2]. In 2019, 465,000 people globally were afflicted with rifampicin-resistant tuberculosis, and about 22% were rifampicin mono-resistant [5].

Extrapulmonary tuberculosis, which accounts for 20% of all tuberculosis cases, is most commonly seen in cervical lymph nodes. And in about 3% of cases of tuberculosis, there is involvement of the axillary lymph nodes [6]. Isolated mediastinal tuberculous lymphadenitis without a parenchymal lung lesion in adults is rare, with an incidence of 0.25-5.8%.

Primary extrapulmonary drug resistance is an uncommon condition, and there is a paucity of data in the medical literature [7]. However, there has been a spike in the notification of the total number of primary drug resistance cases due to the accessibility of health facilities, the availability of state-of-the-art diagnostic labs, and the shortened time lag between initial presentation and treatment starting [8].

Herein, the case of an Indian immunocompetent male is presented who was diagnosed with primary extrapulmonary rifampicin mono-resistant tuberculosis of the cervical, mediastinal, and axillary lymphadenopathy simultaneously without any pulmonary focus.

## Case presentation

A 27-year-old non-diabetic Indian male with no previous history of tuberculosis reported to the outpatient department as a referral case with complaints of swellings in the right side of the neck and right axilla increasing in size over the last 4 months. The swelling was insidious in onset and not associated with any discharge. There was no history of fever, weight loss, loss of appetite, or any night sweats. Also, there was no history of tuberculosis in the family or among acquaintances. He was a private factory worker with no history of substance abuse. Additionally, there was no history of stays in crowded places or imprisonment. Also, there was no history of cancer in the family.

A general examination was suggestive of an endomorphic-built man with a pulse of 81 per minute, a blood pressure of 122/82 mm g, a weight of 71 kg, a temperature of 98.4°F, a peripheral oxygen saturation of 99% in room air, and a respiratory rate of 17 breaths per minute.

The local examination was suggestive of a mobile, firm swelling palpable on pressure from the right deep posterior cervical lymph node, about 1 x 1 cm. Multiple right axillary swellings were palpable, with the largest being 2 x 2 cm in the inferior aspect. The swelling was mobile, firm in consistency, and tender on palpation. However, there were no discharging sinuses or skin discoloration. The axillary (left), submental, and submandibular stations did not include any more palpable nodes. Pallor, icterus, clubbing, cyanosis, or edema were absent. A systematic evaluation turned out to be nothing noteworthy.

**Figure 1:**
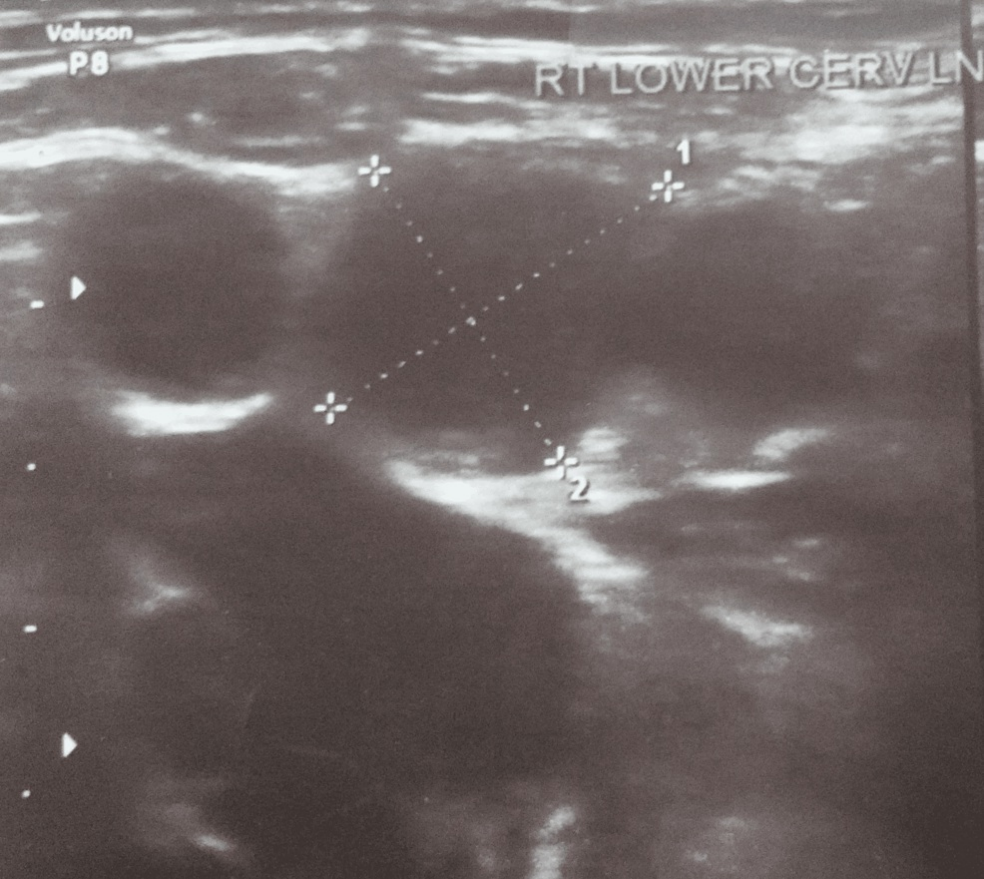
An ultrasound of the neck suggestive of enlarged right cervical lymph nodes.

Hemoglobin of 11.8 g/dl and an elevated erythrocyte sedimentation rate of 51 mm in the first hour were found after a thorough laboratory examination. Every other laboratory parameter (including serum angiotensin-converting enzyme and antigen tests for histoplasma) was within the expected range. He had negative results for hepatitis A, B, and C, as well as non-reactive results for HIV I and II. The skin test for tuberculin showed a 15 x 15 mm induration, which was strongly positive. The induced sputum's smear microscopy and cartridge-based nucleic acid amplification test yielded negative results. The patient's posterior cervical lymph node's cytology was performed by fine-needle aspiration. Histopathology pointed to occasional granulomas composed of epitheloid cells, lymphocytes, and histiocytes in the background of focal necrosis and scanty lymphoid cells. Smear microscopy for acid-fast bacilli was negative. Features were consistent with focal necrotizing granulomatous lymphadenitis. A cartridge-based nucleic acid amplification test from right axillary swelling yielded low *Mycobacterium tuberculosis* with resistance to rifampicin. Simultaneous liquid culture was sterile. Further, an extended drug susceptibility test was unremarkable. His chest radiograph was not suggestive of mediastinal widening or any pulmonary or extrapulmonary lesions. An ultrasound of the neck was suggestive of a few enlarged lymph nodes, with the largest about 6 x 5 mm at level Vb (Figure 1).

**Figure 2:**
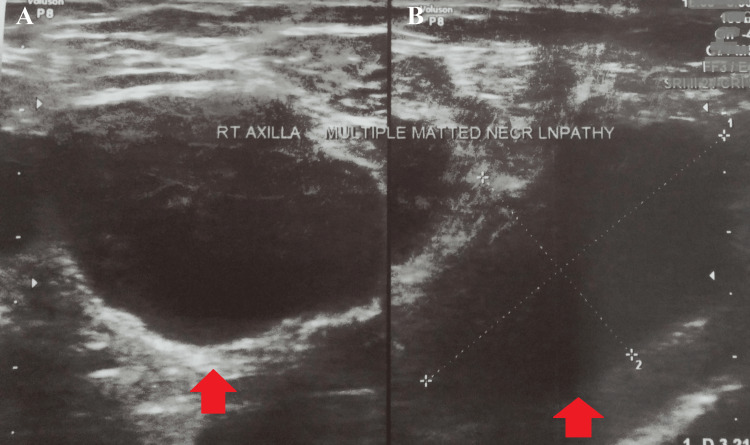
An ultrasound of the right axilla suggestive of a few enlarged axillary lymph nodes in the right axilla. A and B: axillary lymph nodes.

An ultrasound of the axilla was suggestive of a few enlarged axillary lymph nodes in the right axilla in the inferior aspect, the largest measuring 14 x 9 mm in size. Few other lymph nodes were visible, of sizes 6 x 9 mm, 10 x 7 mm, and 6 x 8 mm (Figure 2).

**Figure 3:**
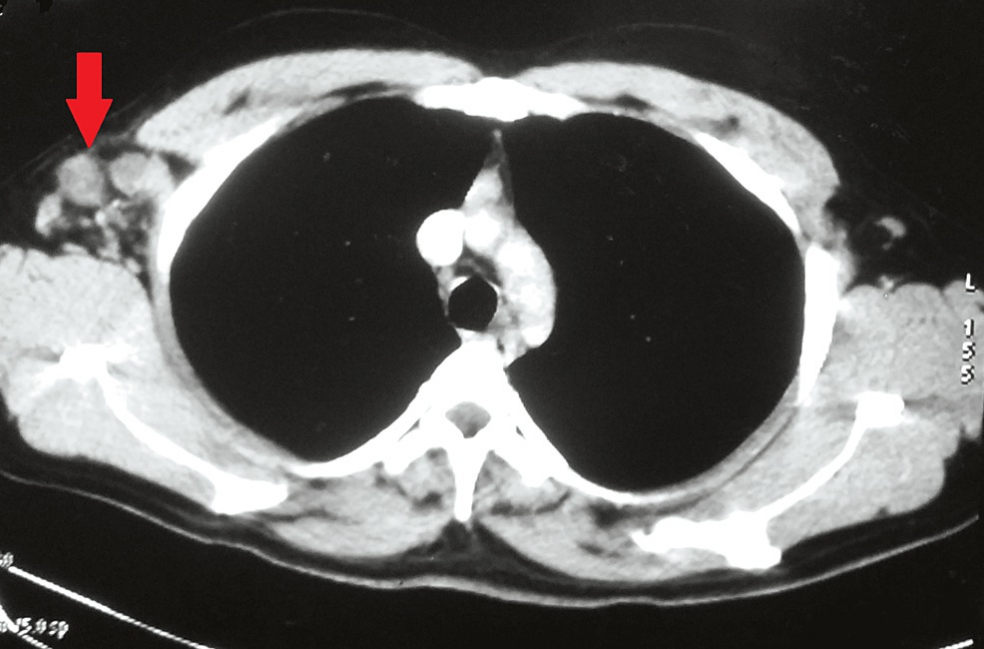
A contrast-enhanced computed tomography of the chest suggestive of multiple enlarged discrete necrotic lymph nodes in the right axillary region.

**Figure 4:**
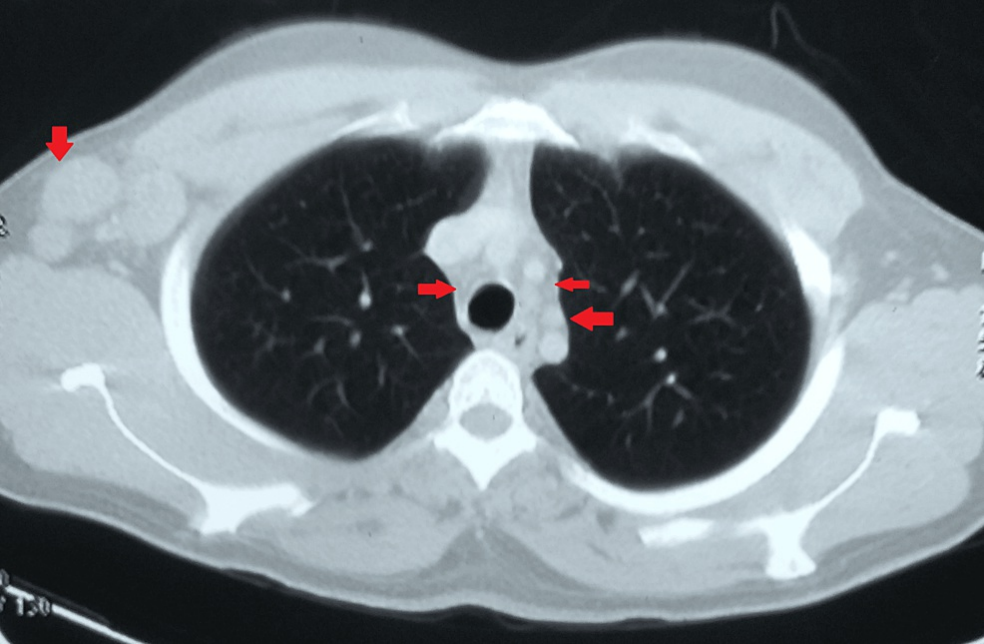
A contrast-enhanced computed tomography of the chest suggestive of multiple borderline mediastinal lymph nodes and right axillary lymph nodes.

A contrast-enhanced computed tomography of the chest was suggestive of multiple enlarged discrete necrotic lymph nodes in the right axillary region, the largest being 28 x 26 mm. Multiple borderline mediastinal lymph nodes were seen, with the largest being 14 x 10 mm in the para-tracheal region (Figures 3,4).

Based on the histopathology, cartridge-based nucleic acid amplification test, radiometric assessment, and laboratory reports, a final diagnosis of primary extrapulmonary rifampicin mono-resistant tuberculosis of the cervical, mediastinal, and axillary lymphadenopathy was made simultaneously without any pulmonary focus, and a pretreatment evaluation was done. As the results of the pretreatment evaluation were within the reference range, he was initiated on an all-oral longer regimen per the national protocol (Table 1).

**Table 1:** An all-oral longer regimen per his weight OD: once daily

Drug	Route of administration	Dose	Duration
Bedaquiline	Per oral	400 mg X OD followed by 200 mg alternate day	Two weeks and then 22 weeks
Linezolid	Per oral	600 mg X OD	6 months
High dose moxifloxacin	Per oral	800 mg X OD	24 months
Cycloserine	Per oral	1000 mg X OD	24 months
Clofazimine	Per oral	200 mg X OD	24 months
Pyridoxine	Per oral	100 mg X OD	24 months

Presently, he has completed 13.5 months of his antituberculous treatment. Largely, his course was unremarkable except for a few minor adverse drug reactions like headaches, vomiting, and nausea, which were taken care of promptly. There is a clinical improvement with a reduction in the size of the cervical and axillary swellings on the follow-up ultrasounds. He is regularly followed up in the outpatient department.

## Discussion

There are several extrapulmonary locations where *Mycobacterium tuberculosis* can manifest, but lymph node tuberculosis is the most frequent [9]. The lymph nodes that are affected by tuberculous disease are the cervical, supraclavicular, axillary, mesenteric, porta hepatis, perihepatic, and inguinal. Females in their early to middle years are more likely to develop axillary tuberculous lymphadenitis. Its greater prevalence on the left side is another noteworthy characteristic. The lymphatic supply of the left upper limb or direct contact with the thoracic duct could be the cause [6].

Mediastinal lymphadenopathy is frequently caused by tuberculosis, sarcoidosis, lymphoma, histoplasmosis, and neoplasia. Differentiating one from the other radiologically could be exceedingly challenging. In tuberculosis, a computed tomography scan of the chest typically indicates right-sided adenopathy and, more precisely, enlargement of the right paratracheal lymph nodes, as seen in the present case [10].

Rifampicin mono-resistance tuberculosis of the lymph nodes is relatively infrequently reported and few studies have reported the prevalence ranging from 2.9-6.5% [11]. Diagnosis of primary rifampicin mono-resistant tuberculosis is mainly based on fine-needle aspiration cytology and identification of the *Mycobacterium tuberculosis* with resistance to rifampicin on the cartridge-based nucleic acid amplification test and culture [12]. Advanced radiometric techniques like computed tomography, magnetic resonance imaging, etc. are helpful in knowing about the details and extent of extrapulmonary tuberculosis sites like deep-seated lymph nodes [13].

Management is essentially conservative after incision and drainage of the infected lymph nodes [14]. The national guidelines of India recommend 540 doses of antituberculous drugs in the all-oral longer regimen [12]. The available information on this condition is scarce. This was just one case, though, and information from multiple locations could be useful in revising the current recommendations for a more focused strategy against rifampicin-resistant lymph node tuberculosis.

## Conclusions

A case of primary rifampicin mono-resistant tuberculosis of the cervical, mediastinal, and axillary lymph nodes has not been documented in the literature. This case emphasizes the urgency of reporting such cases in high-burden settings, as a lack of awareness about the condition could prove lethal to the patients, especially when there is a steady rise in drug resistance in tuberculosis. This case also stresses the need to have a high degree of clinical suspicion in paucibacillary presentations for prompt diagnosis and management.
